# An Infected Urachal Cyst in an Adult Woman

**DOI:** 10.1155/2015/791408

**Published:** 2015-06-18

**Authors:** Serdar Kaya, Besim Haluk Bacanakgıl, Zeynep Soyman, Roya Kerımova, Semiha Battal Havare, Başak Kaya

**Affiliations:** ^1^Istanbul Education and Research Hospital, Department of Obstetrics and Gynecology, Fatih, 34098 Istanbul, Turkey; ^2^Istanbul Education and Research Hospital, Department of Pathology, Fatih, 34098 Istanbul, Turkey; ^3^Kanuni Sultan Süleyman Education and Research Hospital, Department of Obstetrics and Gynecology, Küçükçekmece, 34303 Istanbul, Turkey

## Abstract

The urachus is an embryologic remnant which degenerates after the birth. Defective obliteration of the urachus leads to urachal abnormalities. An infected urachal cyst is one of the urachal abnormalities and this pathology is rare in adult women. We report a case of 33-year-old woman with pelvic pain and dysuria who was diagnosed with infected urachal cyst. Infected urachal cyst is a rare pathology in adult women and this pathology should be considered in the differential diagnosis of acute abdomen.

## 1. Introduction

The urachus is an embryologic remnant which is formed by the obliteration of the allantois. This fibrous tubular structure is located in the midline and extends from the apex of the bladder to the umbilicus. The urachus degenerates after the birth and it is named as median umbilical ligament. The urachal abnormalities are caused by the defective obliteration of the urachus. Urachal abscess is an uncommon disease of the urachal abnormalities and it occurs especially rarely in adults [1, 2]. We report a case of urachal abscess which is originated from urachal cyst in an adult woman.

## 2. Case Presentation

**Figure 1:**
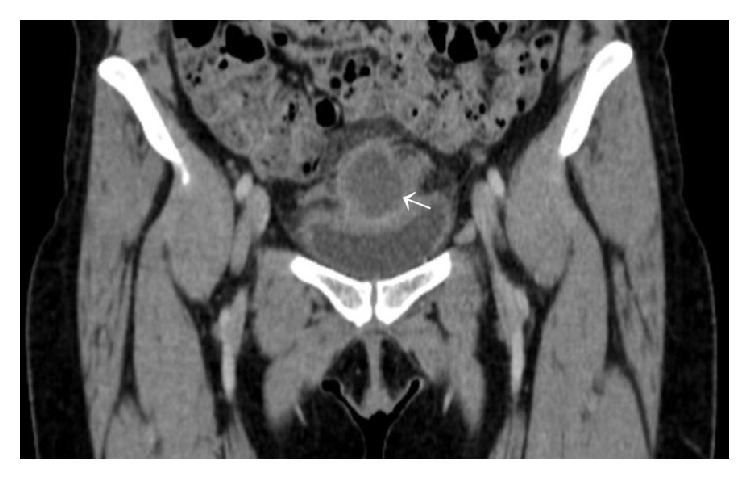
Abdominopelvic computed tomography showing urachal abscess (coronal view).

**Figure 2:**
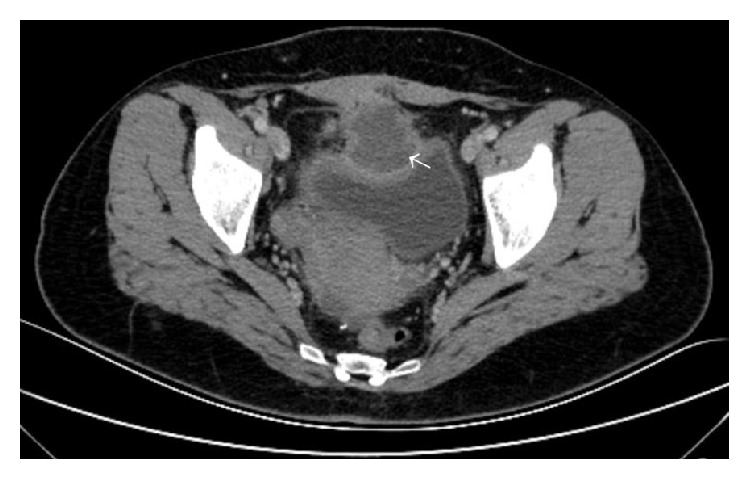
Abdominopelvic computed tomography showing urachal abscess (axial view).

A 33-year-old woman, gravida 2 and para 2, was admitted to the emergency department of our hospital with pelvic pain and dysuria lasting for 3 days. On her physical examination, there was tenderness in the lower abdomen, the body temperature was 38.4°C, the blood pressure was 110/70 mmHg, and the heart rate was 100 beats/min. Laboratory data revealed a white blood cell count of 12.800/*μ*L and a C-reactive protein level of 8.6 mg/dL. Bimanual examination revealed pelvic tenderness and a suprapubic firm mass. Ultrasound examination showed an abscess-like mass (4.5 × 4 × 4 cm in diameter) located at the posterosuperior part of the bladder in the midline. The uterus and adnexa were regular. Abdominopelvic computed tomography revealed a hypodense abscess-like mass in the midline which extended to the rectus muscle and an indentation at the posterior part of the bladder (Figures 1 and 2). The margin of the bladder was regular. A treatment with antibiotics was started before the surgery. A laparotomy was performed via Pfannenstiel incision. Intraoperative observation revealed a semisolid extraperitoneal mass (5 × 6 × 5 cm in diameter) located at the superior part of the bladder. This mass had firm adhesions with anterior abdominal wall and bladder; therefore the mass was extracted with the dome of the bladder. Her postoperative course was uneventful and she was discharged on the sixth day after operation. Pathological examination revealed an infected urachal cyst.

## 3. Discussion

The urachus is an embryonic connection between the bladder dome and the umbilicus, which elongates as the bladder descends. The urachus is obliterated by the fifth to seventh month of gestation and it forms the median umbilical ligament as a fibrous cord which lies between the transversalis fascia and parietal peritoneum [1, 2]. Urachal anomalies are rare in adulthood and are caused by the incomplete obliteration of the urachus. The congenital anomalies of the urachus are patent urachus, urachal sinus, vesicourachal diverticulum, and urachal cyst. Urachal cysts form when both the umbilical and vesical ends of the urachus close while an intervening portion remains patent. These anomalies account for 30% of the congenital urachal anomalies [3]. Urachal cysts usually become symptomatic when these are infected. Infected urachal cysts present with fever, abdominal pain, abdominal tenderness with erythema, lower abdominal mass, nausea, vomiting, and dysuria [4]. The diagnosis of urachal cysts is mainly clinic and the diagnosis is usually confirmed by ultrasonography, computed tomography (CT), and also magnetic resonance imaging (MRI). These imaging methods also give information about the size of cyst and its relationship with peripheral tissue. Ultrasound imaging commonly reveals a tubular mass in the midline below the umbilicus [5]. Although staphylococcal species are usually isolated from the culture of abscess, microorganisms like* Escherichia coli*,* Enterococcus faecium*, and* Klebsiella pneumonia* also can be isolated [6]. The recommended treatment of the urachal abscess is intravenous antibiotic therapy and total surgical excision. The resection of the cyst wall entirely is especially recommended. Because of the high recurrence rate and the risk of malignancy, drainage of the abscess is not recommended. Traditionally surgical excision is performed via laparotomy but laparoscopic excision is also acceptable [7]. Clinical presentation of the urachal abscess may mimic an acute abdomen. Thus in the differential diagnosis other causes of acute abdomen should be primarily considered. Because of urinary symptoms, cystitis and pyelonephritis are also getting involved in differential diagnosis [8]. Although infected urachal cyst is uncommon in adult women, it should be considered in the differential diagnosis of an acute abdomen especially with a mass in the midline. It should be underlined that infected urachal cysts can be misdiagnosed especially as acute appendicitis. The medical history and physical examination of the patient should be detailed.

## References

[1] Ashley R. A., Inman B. A., Routh J. C., Rohlinger A. L., Husmann D. A., Kramer S. A. (2007). Urachal anomalies: a longitudinal study of urachal remnants in children and adults. *Journal of Urology*.

[2] Qureshi K., Maskell D., McMillan C., Wijewardena C. (2013). An infected urachal cyst presenting as an acute abdomen—a case report. *International Journal of Surgery Case Reports*.

[3] Berman S. M., Tolia B. M., Laor E., Reid R. E., Schweizerhof S. P., Freed S. Z. (1988). Urachal remnants in adults. *Urology*.

[4] Allen J. W., Song J., Velcek F. T. (2004). Acute presentation of infected urachal cysts: case report and review of diagnosis and therapeutic interventions. *Pediatric Emergency Care*.

[5] Tazi F., Ahsaini M., Khalouk A. (2012). Abscess of urachal remnants presenting with acute abdomen: a case series. *Journal of Medical Case Reports*.

[6] MacNeily A. E., Koleilat N., Kiruluta H. G., Homsy Y. L. (1992). Urachal abscesses: protean manifestations, their recognition, and management. *Urology*.

[7] Yoo K. H., Lee S.-J., Chang S.-G. (2006). Treatment of infected urachal cysts. *Yonsei Medical Journal*.

[8] Hsu C.-C., Liu Y.-P., Lien W.-C., Lai T.-I., Chen W.-J., Wang H.-P. (2005). Urachal abscess: a cause of adult abdominal pain that cannot be ignored. *American Journal of Emergency Medicine*.

